# Syk-coupled C-type lectins in immunity

**DOI:** 10.1016/j.it.2011.01.002

**Published:** 2011-04

**Authors:** Ann M. Kerrigan, Gordon D. Brown

**Affiliations:** Section of Immunology and Infection, Division of Applied Medicine, Institute of Medical Sciences, University of Aberdeen, Aberdeen, UK

## Abstract

The Syk-coupled C-type lectin receptor Dectin-1 was the first non-Toll like receptor described that could mediate its own intracellular signalling. It was initially identified as important for the innate recognition of and response to fungal pathogens but later studies revealed that it is also involved in triggering adaptive immune responses. It subsequently emerged that Dectin-1 is one of a number of spleen tyrosine kinase-coupled C-type lectin receptors that have been implicated not just in fungal immunity, but also in viral, mycobacterial and helminth infections. Here, we consider the ability of these receptors to trigger different aspects of immunity and highlight their emerging roles in a number of infection scenarios.

## Pattern recognition receptors (PRRs)

The vast body of research in the field of innate immunity over the last 20 years was initially motivated by the prediction that PRRs would recognise evolutionarily conserved molecules on infectious organisms [1]. The subsequent identification of the Toll-like receptors (TLRs) and other PRR families have seen this theory come of age, and our understanding of innate immunity has increased dramatically during this time. We now know that PRR ligands are not unique to pathogens but can be present on commensal microbes, and found endogenously within the host [2]. Many PRRs that detect microbial infection induce innate immune responses by triggering intracellular signalling, which stimulates expression of genes encoding chemokines, cytokines and other immune mediators; and many of these receptors are involved also in controlling the induction of adaptive immunity [2]. TLRs, nucleotide-oligomerization domain (Nod)-like receptors (NLRs), retinoic acid-inducible gene-1 (RIG-1)-like receptors (RLRs) and some C-type lectin receptors (CLRs) are all PRRs that fit within this latter class of receptor that can trigger both innate and adaptive immune responses. Of particular interest to us are CLRs (Box 1) that signal via spleen tyrosine kinase (Syk) to initiate inflammatory responses and function in both innate and adaptive immunity. Of these receptors, an immune function was first demonstrated for Dectin-1, which functions during fungal infection. Other Syk-coupled CLRs subsequently emerged as having roles in fungal immunity; however, recent studies have associated some of these receptors with mycobacterial, viral and helminth infections. The implication of these receptors in a number of infection types, with functions analogous to those of TLRs in terms of their ability to trigger innate and adaptive immune responses, indicates that these Syk-coupled CLRs have a wide significance in immunity. Dectin-1, Dectin-2, Mincle and possibly CLEC5a are all receptors within this category. In this review we summarise these receptors with a particular emphasis on the points raised above; namely, their emerging roles in different types of infection and their ability to trigger both innate and adaptive immunity.

## Signalling via myeloid expressed Syk-coupled C-type lectin receptors

Some Syk-coupled CLRs contain immunoreceptor tyrosine activation (ITAM)-like motifs (also called hemITAMs) in their cytoplasmic tails, whereas others associate with ITAM-containing adaptor molecules, such as Fc receptor γ chain (FcRγ) and DNAX-activating protein of 12 kDa (DAP12). Signalling via ITAMs in myeloid cells is mediated by the recruitment of Syk to phosphorylated tyrosines and several intermediate molecules have been implicated in propagating downstream signalling, which results in various responses including transcriptional regulation through the mitogen-activated protein kinase (MAPK) and nuclear factor of activated T-cells (NFAT) pathways (for a recent review, see [3]). Syk signalling can also activate nuclear factor (NF)-κB and the formation of a complex involving caspase recruitment domain-containing protein 9 (CARD9), B cell lymphoma 10 (Bcl10) mucosa-associated lymphoid tissue lymphoma translocation protein 1 (Malt1) is important for ITAM receptor-mediated triggering of NF-κB and myeloid cell activation [4]. Interestingly CARD9 is not involved in the activation of NF-κB downstream of TLRs and Nod2, although it is involved with their activation of MAPKs [4,5].

## Dectin-1

**Figure 1:**
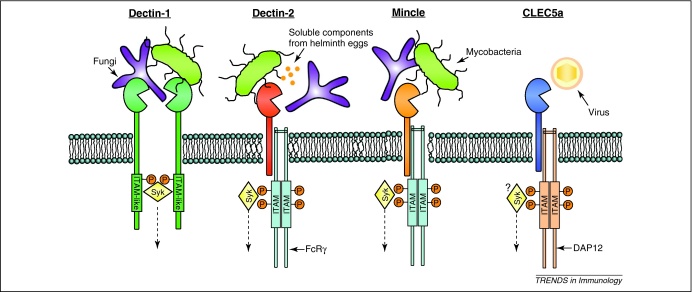
Recognition of microbial ligands by immunorecpetor tyrosine activation motif (ITAM)-coupled C-type lectin receptors (CLRs) leads to intracellular signalling via Syk. The figure shows cartoon representations of Dectin-1, Dectin-2, Mincle and CLEC5a. Dectin-1 recognises fungi and mycobacteria. It contains a cytoplasmic ITAM-like motif and following ligand binding it is thought that Syk is recruited by bridging two monophosphorylated Dectin-1 molecules. Dectin-2 is implicated in the detection of fungi, mycobacteria and helminths. Mincle is implicated in the detection of fungi and mycobacteria. Ligation of both Dectin-2 and Mincle results in phosphorylation of the associated FcRγ chains, recruitment of Syk and activation of downstream signalling. CLEC5a recognises Dengue virions, resulting in the phosphorylation of the associated DAP12 adaptor and downstream signalling, presumably via recruitment of Syk. Broken arrows indicate the occurrence of downstream signalling. It should be noted that for some of these receptors there are also instances of Syk-independent signalling that are not shown here.

Dectin-1 (also known as CLEC7a) is expressed predominantly by myeloid cells and recognises β-glucan carbohydrates in the cell walls of a number of fungal species, as well as unidentified mycobacterial ligand(s) (Figure 1) [6]. In response to β-glucans, Dectin-1 triggers intracellular signalling via a cytoplasmic ITAM-like motif. Downstream signalling pathways induce a number of innate immune responses including recruitment of Syk, activation of NF-κB via CARD9, as well as the activation of MAPKs and NFAT [7–9]; although there are reports of differential use of CARD9 by Dectin-1 in mouse macrophages and dendritic cells (DCs) [10]. Furthermore, recent data appear to directly contradict previous reports that immune responses induced by the β-glucan curdlan (a particulate purified β-glucan) are CARD9-dependent [11]. There is also evidence that Dectin-1 can induce Syk-independent signalling through the kinase Raf-1, which converges with the Syk pathway for synergistic activation of p65, as well as regulation of NF-κB-induced cytokine responses [12]. Given the complexities of Dectin-1 signalling, along with the current ambiguities, further study is required to precisely delineate the downstream pathways in various cell types, particularly the extent of CARD9 involvement.

In addition to innate immune responses, Dectin-1 can trigger adaptive immunity, including Th1, Th17 and cytotoxic T-cell responses (for reviews, see [6,13]). Of particular interest is the ability of Dectin-1 to trigger Th17 responses, which are thought to contribute to fungal clearance (Box 2). The exact mechanism linking Dectin-1-Syk-CARD9 signalling with Th17 responses is unclear, but was shown to depend on regulatory T cells and TGF-β [9]. Furthermore, the tendency of DCs stimulated via Dectin-1 to produce interleukin (IL)-23 as opposed to IL-12 might contribute to limiting Th1 cell differentiation and consequently reduce negative feedback on Th17 cell differentiation [9]. There is evidence suggesting that β-glucans contribute to *Candida*-specific Th17 responses through a collaborative Dectin-1/TLR2 pathway by inducing the production of prostaglandin E2, which in turn up-regulates the Th17 polarising cytokines IL-6 and IL-23 [14].

More recent studies demonstrated that Dectin-1 signalling induced by *C. albicans* and *Aspergillus fumigatus* is required for activation of the Nlrp3 inflammasome and subsequent IL-1β production [15–18], events now known to be crucial for host defence during fungal infection [15,16]. Other advances suggest that Dectin-1 signalling mediates the activation of calcineurin, a protein phosphatase required for the candidacidal activity of neutrophils as well as transcriptional responses to *C. albicans* [19]. Much of this work has pointed towards an antifungal role for Dectin-1 and indeed loss of this PRR in mice has been shown by different groups to result in increased susceptibility to infections with *C. albicans*, *A. fumigatus* and *Pneumocystis carinii* [15,20–23]. Furthermore, a polymorphism in humans, which causes loss of cell surface expression of Dectin-1, rendered individuals susceptible to mucocutaneous infections with *C. albicans*, partly as a consequence of impaired IL-17 production [24].

With regard to mycobacteria, *in vitro* studies implied that recognition by Dectin-1 contributed to uptake, respiratory burst induction, cytokine production and the generation of Th1 and Th17 adaptive responses [25–29]. These findings prompted *in vivo* investigations using an aerosol model of *Mycobacterium tuberculosis* infection in Dectin-1-deficient mice. This work indicated that Dectin-1 might contribute to disease susceptibility but it plays only a minor role in anti-mycobacterial immunity [30].

## Dectin-2

Dectin-2 is expressed on tissue macrophages, some DC subsets and inflammatory monocytes [31]. Its cytoplasmic tail does not contain defined signalling motifs, but it associates with FcRγ to transduce intracellular signalling [32] and the murine form has been shown to bind to several fungi, including *C. albicans* [32–35] (Figure 1). Recent studies demonstrated that Dectin-2 signalling activated NF-κB through the FcRγ-Syk-CARD9 pathway and MAPKs in a Syk-dependent, CARD9-independent fashion [35]. Furthermore, recognition of *C. albicans* α-mannans by Dectin-2 triggered inflammatory responses and Th17 cell differentiation that was important for host defence [11,34,35]. Dectin-2 has been implicated in the recognition of *M. tuberculosis* (Figure 1) [33], although functional studies evaluating its role in mycobacterial infections have not been reported. Furthermore, murine Dectin-2 is the first Syk-coupled CLR to be associated with helminth infections. Its recognition of soluble components derived from the eggs of *Schistosoma mansoni* (Figure 1) triggered the Syk-dependent induction of reactive oxygen species and a potassium efflux activating the Nlrp3 inflammasome [36]. Allergens from house dust mites and fungi are also recognised by Dectin-2, which responds by triggering cysteinyl leukotriene production, suggesting a mechanism whereby clinically relevant allergens can elicit pulmonary inflammation [37].

## Macrophage-inducible C-type lectin (Mincle)

Mincle (also known as Clec4e and Clecsf9) is expressed on macrophages where it can be strongly induced in response to inflammatory stimuli [31]. Mincle itself does not contain any signalling motif but, like Dectin-2, it can associate with FcRγ [38]. Analogous to Dectin-1 and Dectin-2, Mincle has been implicated in the recognition of fungi and mycobacteria, and studies in mice have shown that it contributes to innate inflammatory responses to these microbes in a Syk-dependent manner (Figure 1) [39–43]. Mincle specifically recognises α-mannose residues on *Malassezia* species [40], which are pathogenic fungi that cause skin diseases and fatal sepsis. Mincle also recognises certain strains of *Saccharomyces cerevisiae* and *C. albicans*, although some studies have suggested that it might distinguish structural differences between fungal substrains [39–41]. The detection of mycobacteria by Mincle is via trehalose-6,6-dimycolate (TDM) [42,43], an abundant mycobacterial cell wall glycolipid that is a potent inflammatory virulence factor. TDM and its synthetic analogue trehalose-6,6-dibehenate (TDB), are under investigation for use as adjuvants with recombinant subunit vaccines against tuberculosis. Studies in this area have demonstrated that TDM and TDB selectively activate the FcRγ-Syk-CARD9 pathway to induce protective Th1 and Th17 immunity after subunit vaccination against tuberculosis in mice [44]. The recent work identifying Mincle as a TDM receptor provides a molecular basis for the immunostimulatory activity of TDM and identifies the Mincle-FcRy-Syk-CARD9 pathway as a target for vaccine development against tuberculosis [42,43]. It is notable that the CARD9 pathway was recently found to be essential for resistance to *M. tuberculosis* and it is likely that signalling through Mincle contributes, at least in part, to the generation of these protective host responses [45].

It is important to point out that although the above receptors all trigger Syk-CARD9 signalling, there are fundamental differences in the ways in which they activate Syk. It is thought that Syk is recruited to Dectin-1 by bridging two monophosphorylated molecules [46]. In contrast, Dectin-2 and Mincle engage Syk indirectly through their association with ITAM-containing adaptors, where dually phosphorylated tyrosines are necessary for Syk recruitment. These distinct mechanisms of activating Syk might propagate other distinct signalling events that could ultimately result in differences between cellular responses. Future investigations are likely to reveal whether this is indeed the case.

## C-type lectin domain family 5, member A (CLEC5a)

CLEC5a (also known as myeloid DAP12-associating lectin (MDL-1)), is expressed on the surface of monocytes, macrophages and murine thioglycollate-elicited neutrophils [47,48]. CLEC5a has no defined cytoplasmic signalling motif; however, it interacts with the ITAM-bearing adaptor protein DAP12 [47] as well as the adaptor DAP10 [49]. DAP10 contains a cytoplasmic YINM sequence that facilitates recruitment of phosphatidylinositol 3-kinase (PI3K) and other signalling intermediates after tyrosine phosphorylation, and it is thought to mediate costimulatory signalling in cooperation with DAP12-associated receptors. In contrast to the receptors discussed above, which have been implicated predominantly in fungal and mycobacterial recognition, CLEC5a is the first Syk-coupled CLR shown to directly function as a viral receptor (Figure 1) [50]. The interaction of CLEC5a with dengue virions triggered phosphorylation of the DAP12 ITAM, stimulating the sustained release of proinflammatory cytokines [50], presumably via a Syk-CARD9-dependent pathway, although this was not formally demonstrated. There is no other study concerning CLEC5a and microbial recognition; however, when considered in the context of the previously discussed receptors, it is tempting to speculate that future work could reveal an involvement of CLEC5a in other types of infection and in triggering adaptive immunity.

## Endogenous ligands

It is noteworthy that all the receptors described above have been implicated to various degrees in homeostasis, although Mincle is currently the only concrete example. Mincle can sense necrotic cells through its recognition of SAP130, a nuclear protein released during cellular necrosis; and animal models have demonstrated that this interaction triggered intracellular signalling through the FcRγ-Syk-CARD9 pathway leading to induction of inflammatory cytokines and the recruitment of neutrophils [38]. There are a limited number of studies suggesting that Dectin-1 recognises endogenous ligands. Indeed, Dectin-1 was initially identified as a DC receptor whose recognition of an unidentified ligand on T-cells delivered costimulatory signals resulting in cellular activation and proliferation [51]. Furthermore, the recognition of a ligand on apoptotic cells by Dectin-1 was suggested to be involved in apoptotic cell clearance [52]. Evidence of an endogenous Dectin-2 ligand emerged during investigations of ultraviolet radiation (UV)-induced tolerance, which demonstrated that a soluble Dectin-2 receptor bound to UV induced regulatory T-cells [31]. Furthermore, this study suggested that Dectin-2 and its unidentified T-cell ligand might function during UV-induced immunosupression [31]. Although specific endogenous ligands for Dectin-1 and Dectin-2 have not been discovered, it is likely that future research will lead to their identification and might contribute to the elucidation of the homeostatic functions of these receptors. It will be interesting to learn whether Syk-CARD9 signalling is also a central pathway downstream of the recognition of endogenous ligands. There are suggestions of an endogenous ligand for CLEC5a, which has been implicated in osteoclastogenesis [49]. Interestingly, this study suggested that formation of CLEC5a-DAP12/DAP10 trimolecular complexes is important for this function [49]. Furthermore, antibody-mediated activation of CLEC5a resulted in enhanced recruitment of inflammatory leukocytes to the joint and promotion of bone erosion, and led to the proposal of CLEC5a as a regulator of synovial injury and bone erosion during autoimmune joint inflammation [53].

## Syk-coupled CLRs that function in immunity: an expanding family?

CLEC9a (also known as DNGR-1) and CLEC-2 are Syk-coupled CLRs that have not been specifically implicated in microbial recognition. Like Dectin-1, these receptors contain cytoplasmic ITAM-like motifs through which they mediate Syk-dependent signalling. CLEC-2 is expressed on platelets and mouse neutrophils [54,55] and its recognition of the snake venom toxin rhodocytin mediates Syk-dependent platelet activation [54]. CLEC-2 has also been implicated as an HIV-1 attachment factor; however, binding of the virus to CLEC-2 was via (an) endogenous host factor(s) incorporated into the viral envelope [56]. The transmembrane protein podoplanin was identified as an endogenous CLEC-2 ligand and induced platelet activation in a CLEC-2 and Syk-dependent manner [57,58]. Furthermore, recent studies in mice have demonstrated that the activation of CLEC-2 by podoplanin facilitates the separation of lymphatic and blood vasculatures during development [59,60]. Less is known about CLEC9a, which is expressed on a subset of DCs (CD8α^+^ DCs in mice and their BDCA2^+^ equivalents in humans) [61–63]. This receptor recognises an unidentified ligand on necrotic cells, and can mediate Syk-dependent cross presentation of dead cell-associated antigens [63]. As Dectin-1, Dectin-2, Mincle and CLEC5a have all been shown to have microbial ligands, as well as implicated, to various degrees, in the recognition of endogenous ligands, it will be interesting to see whether future work will continue the trend with the identification of microbial ligands for CLEC9a and CLEC-2.

## Concluding remarks

Recent studies have highlighted the growing relevance of myeloid expressed Syk-coupled CLRs in antimicrobial immunity. In this review, we have highlighted these receptors and summarised their functions in a number of infection types. Signalling via the Syk-CARD9 pathway is a key underlying feature common to these receptors; however, their signalling pathways are complex and also feature Syk-independent and CARD9-independent mechanisms, as well as differential signalling between cell types. Their capacity to drive inflammation is important for innate immunity; and their ability to induce adaptive immunity appears to be crucial for host defence. It will be interesting to see whether the inflammatory and Th17 responses driven by these receptors can also result in pathology and autoimmune disease. Thus, focusing on understanding the mechanisms that regulate these responses is likely to have important future therapeutic implications.

## Box 1 C-type lectin receptors

The C-type lectin receptors (CLRs) are a large superfamily of proteins characterised by the presence of one or more C-type lectin-like domains (CTLDs). The superfamily has been divided into 17 groups on the basis of phylogeny and domain organisation [64]. CLRs were originally described as Ca^2+^-dependent, carbohydrate-binding proteins; however, many CLRs do not bind carbohydrate ligands and, furthermore, display Ca^2+^-independent ligand binding. [64]. Despite the presence of a highly conserved domain, CLRs are functionally diverse and have been implicated in various processes, including cell adhesion, tissue integration and remodelling, platelet activation, complement activation, pathogen recognition, endocytosis and phagocytosis. A prominent role for CLRs in immunity was originally identified in natural killer cells, where they were implicated in controlling natural killer cell function in response to transformed and virally infected cells. The identification of CLRs on myeloid cells raised the possibility that they serve similar functions in controlling cellular activation. Of relevance to this review are myeloid-expressed CLRs belonging to the Group II and V subgroups, many of which have been implicated in immunity and homeostasis. These CLRs are all type II transmembrane receptors with a single extracellular CTLD. In all instances, their functions have been linked to the presence of signalling motifs in their cytoplasmic tails, or their ability to couple with signalling chains, which trigger intracellular signalling through various pathways (for recent reviews on signalling mechanisms, see [3,65]).

## Box 2 Th17 cells in host defence and autoimmunity

T helper cells that were initially characterised by the production of IL-17A (IL-17) have recently emerged as a third distinct subset of effector T cells, the differentiation of which involves cytokines, including TGF-β, IL-1β, IL-6, IL-21 and IL-23, and transcription factors, including retinoic acid receptor-related orphan nuclear receptors (ROR)α and RORγt [66]. Although the differentiation of this subset has been the focus of intensive research, the precise involvement of cytokines such as IL-23 and TGF-β has presented some controversies, and the relatedness of human and mouse Th17 cells has been questioned.

Th17 cells produce a variety of cytokines, such as IL-17, IL-17F, IL-21 and IL-22, which are involved in initiating inflammatory responses. As a result of this activity, Th17 responses have been investigated in terms of their contribution to anti-microbial responses and the induction of autoimmunity. In the case of fungi, there has been some controversy but a number of investigations have shown that Th17 responses are required for protection, at least at mucosal sites [67]. Indeed, patients with genetic defects resulting in aberrant Th17 responses, including Dectin-1 and CARD9 deficiencies, suffer from chronic mucocutaneous candidiasis and other mucosal fungal infections [24,68]. Th17 responses have also been implicated in anti-bacterial immune responses; however, their role in viral and parasitic infections is less clear [69].

Growing evidence has revealed the role of Th17 responses in pathology and autoimmune disease. IL-17, IL-17F and Th17 cells have been implicated in autoimmune diseases, including rheumatoid arthritis, psoriasis, inflammatory bowel disease, multiple sclerosis, systemic lupus erythematosus and asthma [70]. These developments have led to increased efforts in defining the precise involvement of Th17 cells in antimicrobial responses and in the induction of autoimmunity; and in developing strategies to block their pathological effects.
